# T cells and their products in diabetic kidney disease

**DOI:** 10.3389/fimmu.2023.1084448

**Published:** 2023-01-26

**Authors:** Yue Liu, Yaodong Lv, Tingwei Zhang, Tongtong Huang, Yating Lang, Qinghao Sheng, Yingxiao Liu, Zhijuan Kong, Ying Gao, Shangwei Lu, Meilin Yang, Yaqi Luan, Xining Wang, Zhimei Lv

**Affiliations:** ^1^ Department of Nephrology, Shandong Provincial Hospital Affiliated to Shandong First Medical University, Jinan, China; ^2^ Department of Neurology, Yantai Yuhuangding Hospital, Shandong University, Yantai, China

**Keywords:** diabetic kidney disease, T cells, cytokines, immunologic function, differentiation, recruitment, therapeutic methods

## Abstract

Diabetic kidney disease (DKD) is the most common cause of end-stage renal disease and has gradually become a public health problem worldwide. DKD is increasingly recognized as a comprehensive inflammatory disease that is largely regulated by T cells. Given the pivotal role of T cells and T cells-producing cytokines in DKD, we summarized recent advances concerning T cells in the progression of type 2 diabetic nephropathy and provided a novel perspective of immune-related factors in diabetes. Specific emphasis is placed on the classification of T cells, process of T cell recruitment, function of T cells in the development of diabetic kidney damage, and potential treatments and therapeutic strategies involving T cells.

## 1 Introduction

Diabetic kidney disease (DKD) is a highly prevalent microvascular complication of diabetes that affects >50% of incident cases of diabetes mellitus (DM) and profoundly contributes to patient morbidity and mortality (1). Clinically, DKD is characterized by the presence of albuminuria and decreased estimated glomerular filtration. DKD is diagnosed based on glomerular basement membrane thickening, mesangial expansion, diffuse or nodular glomerulosclerosis, podocyte loss, and interstitial fibrosis on pathology and histology (2). Multiple mechanisms contribute to the outcome of DKD. Among them, nonimmune factors, metabolism, and hemodynamics are considered the most crucial causes of renal damage in patients with type 2 DM (T2DM) and DKD in traditional perceptions (3–5). Therefore, optimal control of hyperglycemia and intensive treatments for elevated blood pressure remain the current management strategies for patients with diabetes. However, current treatments are insufficient to prevent its progression in a large proportion of patients, and the prevalence of DKD is still increasing every year. The mechanisms leading to the development of renal dysfunction in diabetes are not fully understood; therefore, there is an urgent need to identify the pathogenesis and therapeutic approaches to DKD.

In comparison to merely considering DKD a non-immune metabolic disease induced by hyperglycemia, current studies emphasize that DKD is also an inflammatory disease (6, 7). The infiltration of immune cells, which is related to innate and adaptive immunity, may be involved in hyperglycemia-induced renal injury (8). In particular, the role of T-cells in the development of DKD has been confirmed (9). On the one hand, high glucose has been verified to induce T cells recruitment, activation, differentiation, and maturation, even the cytokine factor expression profiles of T cell (10). On the other hand, serum concentrations of chemokines and cytokines produced by T cells have been assessed in patients with diabetes, which are supposedly to predict the onset of diabetic complications.

Hence, we summarized the updated progress in the aspects of differentiation, recruitment, function of T cells, and their products in the DKD as well as the potential strategies for the treatment of DKD, hoping to provide insights for future research.

## 2 Classification and differentiation of T cells in diabetic kidney disease

T cells are involved in host defense and clearance of pathogens. In general, T cells are divided into two species according to their constitutive chains, called “conventional T cells” and “unconventional T cells,” which operate in utterly different ways to regulate and coordinate immune responses in the kidney.

Classically, T cells that express T-cell receptors (TCRs) with α- and β-chains are classified as conventional T cells; specifically, conventional T cells can be separated into CD8^+^ T cells and CD4^+^ T cells, and these cells recognize peptides presented by the major histocompatibility complex class II and I. Based on the specific function, the differentiated CD4^+^ T cell subsets were further distinguished into T-helper (Th) cells and regulatory T cells (Tregs).

According to previous research, unconventional T cells recognize antigens in the absence of classical restriction *via* the major histocompatibility complex and respond rapidly upon antigen encounters (11). In the kidneys, unconventional T cells include mucosal-associated invariant T (MAIT), natural killer T (NKT), and γδT cells (12).

In addition, tissue-resident memory (TRM) T-cells, the most abundant memory T-cell subset, have been identified as a class of T cells that reside in the kidney (13, 14). The different phenotypes and functions of TRM are derived from its position in various tissues (15). Due to the synergistic effect of the anatomical localization effector and memory phenotype, TRM T-cells located in the kidney are critically involved in DKDs.

### 2.1 Overview of T helper cells

Th cells are a cluster of highly plastic CD4^+^ T cells and are simultaneously important contributors to the autoimmunity and inflammation induced by DKD. Many modulatory mechanisms employed by Th cells contribute to the adjustment of renal tissue damage, such as by mediating the production of local cytokines. According to their cytokine and transcription factor expression profiles, Th cells are primarily grouped into Th1, Th2, Th3, Th9, Th17, Th22, T follicular helper (Tfh), and Tregs.

As a flock of plastic cells, Th cell subsets can acquire regulatory functions upon chronic stimulation in diabetes, opening a new perspective for the exploration of immunomodulatory mechanisms for diabetes (8, 16). Hence, the classification and differentiation of Th cells in diabetes and its renal complications are associated with their unique subsets, which are described in the following sections.

#### 2.1.1 Th1

Since 1986, a groundbreaking study has elaborated the patterns of lymphokine activity production of Th1 and Th2 cells; Th1 expresses its signature cytokines such as interleukin (IL)-2, interferon-γ (IFN-γ), tumor necrosis factor-α (TNF-α), and transcription factor T-box (T-bet), among others (17–19), owing to which it participates in the activation of macrophage cell-mediated immunity and systematically regulates cellular function.

Notably, the level of Th1 can be mediated by multiple factors. For example, STAT4 and STAT1, members of the signal transducer and activator of transcription (STAT) family, are crucial for inducing differentiation and maintaining the Th1 cell phenotype (20, 21). T-bet also modifies the level of Th1 by activating STAT1 (22). In contrast, the cell immunoglobulin domain and mucin domain 3 are extensively considered suppressants of IFN-γ-producing T-cells (23).

Meanwhile, Th1 has been shown to respond to preceding and accompanying immunoreaction in DM (24). In clinical settings, Th1 cells dramatically increase in patients with type 2 diabetic nephropathy (T2DN), and the degree of proteinuria is positively correlated with aberrant cytokine production, such as IFN-γ and IL-2R (25). Creatinine clearance is also negatively correlated with plasma TNF-α and urinary MCP-1 levels.

#### 2.1.2 Th2

Th2 and its produced IL-4, IL-5, IL-9, IL-10 and IL-13 are related to the pathogenesis of DKD (26, 27). Furthermore, the inherent link between Th1 and Th2 has been discussed in the immunopathogenesis of diabetes. In contrast, IL-10 and IL-4 produced by Th2 can dampen IFN-γ secretion and suppress Th1 cell activation (in the regulation of humoral immunity, among other processes (27, 28).

Contrastingly, the decrease in T-bet produced by Th1 corresponds with the increase in plasma IL-4 secreted by Th2, implying an imbalance between Th1 and Th2 (29). Hence, upregulating GATA-3 and IL-4 expression and downregulating T-bet and IFN-γ levels may provide a novel therapeutic method for type 1 diabetes (T1D) treatment in non-obese diabetic (NOD) mice (30). Intriguingly, GATA-3, a promoter of Th2 responses, was increased in diabetes (31, 32), while peritoneal dialysis may increase the frequency of Th2 cells during the treatment of DKD (33).

#### 2.1.3 Th17

As discovered in 2005, Th17 secrete IL-17 as its signature cytokine (34). After Th17 cells receive active signaling, the JAK/STAT pathway directly culminates in activation of STAT3 of RORγT, resulting in the production of IL-17 (35). In contrast, IL-2-induced activation of STAT5 causes a decrease in ROR-γt and a transient downregulation of IL-17 (36). In addition to ROR-γt, the differentiation of Th17 cells can be directed by transforming growth factor (TGF)-β, IL-6, IL-1β, and IL-23 (37, 38). Interestingly, unique cytokines can induce different types of Th17 cells. For example, the proinflammatory subtype of Th17 cells is induced by TGF-β, whereas the less pathogenic subtype is promoted by IL-1β (36, 39). In peripheral blood lymphocytes from patients with diabetes, promoter activation was verified as the core principle of the change in IL-17 and its downstream signaling (40).

On the immune-mediated kidney disease, Th17 cells are likely to get upregulated in DKD, resulting in a general increase of IFN-γ and IL-17A in streptozotocin (STZ)-induced diabetes (41). A clinical study based on blood samples collected from 56 patients with nephropathy and 57 patients with diabetes revealed that patients carrying at least one allele of the IL-17A *(rs2275913)* gene polymorphism were vulnerable to DKDs (42). In a cross-sectional study, the level of serum IL-17 was also found to be lower in individuals with diabetes or renal lesions in Asian and Indian populations (43).

Furthermore, in terms of DKD treatment, IL-17A gradually demonstrates dose-dependent properties. As mentioned above, presence of IL-17A in individuals with diabetes and diabetic mouse models is an obvious characteristic, and serum and urinary levels of IL-17A in the former with advanced DKD confirms this finding; additionally, low doses of IL-17A have a noteworthy therapeutic effect on podocytes and tubular cells (44). The protective effect of IL-17 may also be dependent on its subsets, as low doses of IL-17A and IL-17F can prevent severe impairment of renal function at the beginning of the course of DKD; however, IL-17C or IL-17E do not show a similar effect (40).

With respect to the relative ratio of Th17 cells, interesting studies have demonstrated that the Th17/Treg ratio promotes inflammation and may hasten the development of diabetic complications. The increase in Th17 or decrease in Tregs may be a contributing factor to the deterioration of kidney function (45, 46). The Th17/Th1 response ratio is a potential contributor to β cell destruction and provides a novel biomarker for the rapid diagnosis of T1D preceding the clinical end. Moreover, similar investigations have been performed on the serum levels of relevant cytokines in patients with T2DM, and the Th1/Th2/Th17/Treg paradigm has been demonstrated to skew toward Th1 and Th17 (26).

#### 2.1.4 Th3, Th9, and Th22

As the research has progressed, various types of Th cells have been discovered to be involved in diabetic complications. Characterized by high expression of TGF-β, Th3 has a negative correlation with DKD at the onset of the disease rather than in the prediabetic phase (47). Another subset, named Th9 cells, is designated as IL-9 producers. With the technical support of nanoscale flow cytometry, Semenchuk et al. found that IL-9 is inversely related to the quantification of urinary podocyte-derived extracellular vesicles (48). Additionally, Th22 participates in the regulation of DKD by producing IL-22 (49).

### 2.2 Tfh cells

Tfh cells are another distinctive Th subset of cells that require the synergistic action of IL-6 and IL-21 to drive differentiation (50, 51). Tfh cells are involved in diabetic syndrome, leading to elevated levels of CXCR5, ICOS, PDCD1, BCL6, and IL21 (52). Many subsets of Tfh cells, such as CXCR5^+^ PD1^+^ ICOS^+^ and CD4^+^ CXCR5^+^ PD-1^+^, are increased in children and adults with diabetes (53, 54). In particular, CD4^+^ CXCR5^+^ Tfh cells have been confirmed to manipulate the levels of estimated glomerular filtration rate (GFR), creatinine, urea, urinary protein, fasting and postprandial blood glucose, and hemoglobin A1c in patients with DKD (55).

### 2.3 Regulatory T cells

Analysis of gene polymorphisms revealed that FOXP3^+^ Tregs were reduced in patients at the onset of diabetes (56). The apoptosis of Tregs is affected by aberrant IL-2R signaling, leading to a decrease in FOXP3 persistence and impacting the establishment of tolerance (57). Therefore, a single infusion of autologous polyclonal Tregs and recombinant human low-dose IL-2 may be a novel treatment for diabetes (58).

In addition to suppressing T cells, NK cells, NKT cells, B cells, and dendritic cells in the adaptive immune responses, Treg cells play a fundamental role in the pathological development of DN, maintaining a dynamic equilibrium between inflammatory cytokines and anti-inflammatory cytokines (59–61). Treg cells can control phenotypic changes by increasing (IFN-γ, IL-2, and IL-17) and decreasing (IL-10, IL-35, and TGF-β) the levels of anti-inflammatory cytokines (62, 63). Generally, the population and function of Tregs has a peculiar effect on immunoregulation in patients with diabetes.

### 2.4 CD8^+^ T cells

Recent findings have reported that CD8^+^ T cells were increased in patients with diabetes and that suppressing CD8^+^ T cells may alleviate the pathological reaction of DKD (64). Furthermore, infiltration of CD8^+^ effector T cells is important for recruiting macrophages to ameliorate systemic insulin resistance in mice fed a high-fat diet (65). Interestingly, the proportion of CD8^+^ TRM cells was increased in DKD and further promoted podocyte injury and glomerulosclerosis, suggesting a pivotal role of CD8^+^ T cells in podocyte damage in insulin-resistant patients with DN (66).

### 2.5 NKT cells and γδ T Cells

NKT cells are another characteristic T-cell subset that links the innate and adaptive immune systems, and abnormalities in the frequency and activity of NKT cells may be attributed to the exacerbation of T1D (67). NKT cells play a fundamental role in various renal diseases involving abnormal metabolism. For instance, inappropriate overactivation of NKT cells can cause kidney damage *via* the TNF-α/Fas ligand pathway (68). In progressive non-alcoholic fatty liver disease, NKT cells also cause glomerular function and renal immunotoxicity (69). Furthermore, during chronic kidney disease (CKD) progression, the raise of CD3^-^ CD56^+^ NK cells were observed in tubulointerstitial, and the frequency of CD3^-^ CD56^+^ NK cells and CD3^+^ CD56^+^ NKT cells were also remarkably elevated in the peripheral blood of diabetic patients (70, 71). Simultaneously, NKT cells express IL-4, IFN-γ, natural-killer group 2 member D, and IL-17, thus inducing vascular injuries (72).

The subsets of yδ TCR^+^ cells, such as CD27^-^ CD44hi and CD27^+^ CD44lo, have also been increased in prediabetic NOD mice; however, the knowledge of concrete mechanism of γδ T in DKDs has been limited until now (73).

### 2.6 Mucosal-associated invariant T cells

MAIT cells not only belong to a specialized subset of unconventional (non-major histocompatibility complex-restricted) T cells but have also emerged as key players in immunity and pathological inflammation. First, human MAIT cells express a semi-invariant TCRa chain (Va7.2, coupled with restricted Ja segments), coexpressed with high levels of the C-type lectin receptor CD161, which is beneficial to its presentation in human barrier sites such as the kidneys (74, 75). Moreover, MAIT cells were reported to sharply increase with a cytokine cocktail comprising IL-12, IL-15, and IL-18, which participates in the progression of chronic inflammation (76).

Furthermore, researchers have found that dysregulation of MAIT cells may influence the severity of insulin resistance. The frequency of MAIT has been shown to be influenced by BMI, and there is a positive correlation between MAIT and HbA1c levels, accompanied by an increase in CD25 and CD69 (77, 78). Another study by Harms et al. observed a significant increase in the CD27- MAIT cell subset and IL-17A in patients with T1DM, particularly in younger patients (77).

### 2.7 Tissue-resident memory T-cells

CD69^+^ CD103^+^ and CD69^+^ CD103^-^ TRM cells have been identified as two primary subsets of TRM cells (15). CD69 binds to S1PR1 on the T cell membrane, restraining the migration of memory T cells from the blood to peripheral tissues (79). Therefore, a mass of TRM-T cells exists in the kidney rather than in the circulation. After encountering antigens *in vivo* and *in vitro*, native T cells rapidly produce effector T cells and swiftly migrate to lymphoid and non-lymphoid tissues, persisting through barrier tissues, such as the kidney (80, 81). Following inflammation resolution, antigen-specific effector T cells differentiate into diverse memory T cell subsets with distinct trafficking properties.

In the tubulointerstitium of DKD, a recent xCell analysis has identified immune cells, thus revealing significant changes, including activated Th2 cells, CD4^+^ T cells, CD8^+^ T cells, dendritic cells, conventional dendritic cells, M1 macrophages, and restrained Tregs (82). However, knowledge of T cell differentiation in DKD remains limited.

## 3 Recruitment and activation of T cells in diabetic kidney disease

As early as 2012, the aberrant recruitment and activation of T cells in DKD had been discussed (83). The results showed an increase in CD4^+^, CD8^+^, and CD20^+^ cells in the interstitium, indicating that aberrant intrarenal infiltration and recruitment of T cells are potential immunopathological mechanisms of diabetic kidney lesions. Immunohistochemical analysis also showed that a substantial proportion of juxtaglomerular apparatuses in patients with T1DM contained abundant T cells (84).

To exert their local effects on renal injury, circulating T cells must reach the site of inflammation. Typically, some T cells, such as Th cells, do not possess a residency status similar to that of other immune cells, such as kidney TRM-T cells.

A series of tissue-specific markers has been reported to activate T cells in the kidney. Once activated, T cells can expand their immunoreaction, inducing chemokine release and more widespread recruitment of T cells (85). However, little is known about the trafficking of T cells into the kidney under hyperglycemic conditions, and their migration patterns have been the subject of extensive studies (17). Hence, the methods for circulating T cell migration into kidney should be assessed in the next step of research.

### 3.1 Chemokines and its receptor

There is a positive feedback between chemokines and T cells in the inflammatory response and immune adjustment. In other words, chemokines facilitate the attraction of circulating T-cells and stimulate their infiltration into tissues. T cells also participate in the regulation of the pathophysiological progression of renal insufficiency by producing chemokines. In this section, we focus on the chemokines involved in the recruitment of T cells in DKD.

#### 3.1.1 CXCL9-CXCR3

Multiple studies have shown that the urinary level of C-X-C motif chemokine ligand 9 (CXCL9) mRNA is significantly elevated and correlated with eGFR decline, which can be utilized to measure and stratify the risk of DKD (86, 87).

On the other side of the CXCL9-CXCR3 axis, C-X-C motif chemokine receptor 3 (CXCR3) is a well-known chemokine receptor predominantly expressed on the surface of Th1 polarized T cells and regulates the recruitment of Th1 cells (88). Moreover, CXCL9 and CXCR3 have been found to be influenced by advanced glycosylation end products (AGEs), implying that Th1 can be recruited under diabetic conditions (89).

#### 3.1.2 CXCL10/CXCR3

In an exploratory study, the CXCL10/CXCR3 axis was observed in the autoimmune process in T1D. Serum levels of CXCL10, a well-known Th1 chemokine, are elevated in patients with T1D, suggesting that CXCL10 plays a critical role in predicting T1D (90). Most importantly, CXCL10 may be induced by IFN-γ, promoting T cell infiltration and accelerating beta cell destruction (91).

#### 3.1.3 CXCR5

In individuals with DN, the increase in CD4^+^ CXCR5^+^ Tfh cells may significantly increase creatinine, urea, urinary protein levels, fasting blood glucose, postprandial blood glucose, and HbA1c and decrease estimated GFR (55). In the future, the increased number of CD4^+^ CXCR5^+^ PD-1^+^ Tfh cells in patients with DN may be a new target for intervention in DKD (47).

#### 3.1.4 CX3CL1-CX3CR1

At an early stage of nephropathy, CX3CR1^+^ T cells are elevated and induce IL-17A production in renal impairment (92–94). In addition, the polarization of TH17 or Treg cells may be associated with an increase in CX3CR1 reporter gene expression in T cells (92). Several studies have shown that CX3CR1 and CX3CL1 are upregulated in the kidneys of patients with diabetes, accompanied by an increase in urea, creatinine, A/C ratio, HbA1C, and IgG; however, the concrete mechanism of CX3CL1-CX3CR1 recruiting T cells requires further exploration in DKD (93).

#### 3.1.5 CCL5 (RANTES)- CCR5

CCL5 is a β-chemokine, which is also known as RANTES (regulated on activation, normal T cell expressed and secreted), and can function as a chemotactic factor for T cells and induce cellular activation of normal T cells (95) In inflammatory kidney diseases, constitutive RANTES expression facilitates the accumulation of CD4+ T cells in the kidney, while the administration of RANTES-neutralizing antibody is helpful in reducing the accumulation of T cells in the kidneys to a large degree. Moreover, RANTES-neutralizing antibodies can reduce the deposition of collagen in obstructed kidneys (96).

There is no doubt that CCR5 is a characteristic of Th1 lymphocytes and a critical chemokine receptor for trafficking of TH1 cells to the kidney (88, 97); however, the status of CCR5 in T2DM and microvascular complications remains controversial. The problems are mainly focused on the significant discrepancy in the allelic frequency of CCR5 between different ethnic groups. In Asian populations and people with T2DM, the CCR5 *59029G/A* polymorphism is significantly associated with an enhanced susceptibility to DN (98). Nevertheless, the CCR5 *59029 A* allele only has a convincing association with nephropathy in T2DM Malaysian Chinese population but is weakly associated with nephropathy in Malaysian Indian population (99). Additionally, in native Estonian patients with T2D, there was a lack of association between the C*CR5-Δ32* mutation and DKDs (100). Hence, further research is needed to determine whether CCR5 is associated with DKD worldwide.

#### 3.1.6 CCL2 (MCP-1)- CCR2

Chemokine ligand 2 (CCL2) binds to its receptor, C-C chemokine receptor 2 (CCR2), initiating the migration and infiltration of T cells and regulating tissue inflammation (101). A longitudinal analysis followed the fate of CCR2^−/−^ T cells and observed that CCR2 regulates the immune response by modulating the effector/regulatory T ratio. Additionally, CCR2 deficiency in T cells decreases the levels of Th17 cells while promoting a program that induces the accumulation of Foxp3+ Tregs *in vivo (* 102).

Recent studies have suggested that CCL2 (MCP-1) is a key chemokine involved in DN. In a blood sample analysis conducted in Iran, CCL2 was gradually elevated in patients with T1D with disease duration (103). Furthermore, the blockade of this pathway plays a protective role in insulin resistance, modulation of adipose tissue, restoration of renal function, and restraint of progressive fibrosis in hyperglycemic kidneys (104, 105). A phase Ia study targeting emapticap impeded the CCL2/CCR2 receptor axis and exerted beneficial effects on ACR and HbAlc in albuminuric T2D (106). Overall, the CCL2/CCR2 receptor axis is thought to be crucial for the progression of DKD.

#### 3.1.7 Interleukins

T cells not only produce several members of the IL family but are also recruited by other immunocytes produced ILs, such as IL-18, IL-19, among others.

IL-18 is not mainly produced by T cells but plays an underlying pathophysiological role in the progression of T cell differentiation in DKD. IL-18 induces plasticity in established Th1 and Th2 cells (107–109). It also acts synergistically with IL-12 to increase the level of IFN-γ, a Th1 cytokine (110). In recent years, a cross-sectional study of patients with T2D showed that IL-18 levels were significantly boosted at a low eGFR and positively correlated with the development of DN and urinary albumin excretion (UAE) rate (111, 112).

Similarly, IL-19 were markedly positively correlated with Hs-CRP, cystatin C, and UAE in patients with DN (113). The reduction in IL-19 levels contributes to the suppression of T-cell responses and inhibition of the regulatory activity of CD4^+^ T cells, causing cell-mediated immunosuppression (114). Therefore, IL-19 may be another target for regulating T cell differentiation in DKD.

#### 3.1.8 TGF-β

In renal inflammatory diseases, TGF-β has been demonstrated to orchestrate the differentiation of T cells, including Th17 and Foxp3^+^ Treg cells (96) Additionally, rats with hyperglycemia-induced microalbuminuria possess upregulated TGF-β and serum creatinine levels (115). Recently, the role of TGF-β in promoting the characterized T cell cytokines, IL-9 and IL-17, has become more widely accepted. TGF-β controls the secretion of both these cytokines, subsequently mediating fasting and postprandial glucose and HbAlc levels in patients with DN (116). Taken together, restraining TGF-β may be considered as an approach aimed at attenuating T1D in the immediate future.

### 3.2 Other factors that regulate T cells

Similar to chemokines, there are many other factors that facilitate the assembly and infiltration of T cells, such as C3a and its receptor, AGE, KIM-1, Chromogranin A, among others.

#### 3.2.1 Complement C3a and its receptor

Emerging evidence suggests that the expression of C3a and C3aR is involved in DN pathogenesis (117). Compared with normal controls, C3aR was significantly increased in the renal specimens of patients with diabetes and wild-type (WT) diabetic mice. *In vitro* microarray profiling revealed the underlying mechanism that C3a plays a role in suppressing T-cell adaptive immunity by interfering with CD4^+^ and CD8^+^ T cell infiltration, and in an *in vitro* study, C3a was able to enhance differentiation of the T-cell lineage in inflammatory responses (118). Thus, C3aR may be a promising target for T cell recruitment and activation.

#### 3.2.2 Advanced glycosylation end products

In peripheral blood T lymphocytes, the expression of AGE binding sites serves to target T cells to the AGE-rich renal tissues. With the increase and accumulation of AGE products and AGE-modified proteins, their binding to the AGE receptor on T cells is remarkably increased, promoting the synthesis and release of proinflammatory cytokines in diabetes (119).

#### 3.2.3 KIM-1

KIM-1 is also known as T-cell lg mucin 1 (TIM-1) or hepatitis A virus cellular receptor 1 and has been reported as a transmembrane glycoprotein receptor on T cells (120). Recent studies have revealed elevations in KIM-1, suggesting that glycemic variations may increase the production of KIM-1 in CD8^+^ T cells in individuals with DKD, thereby increasing the risk of DKD (121). The elevations in circulating KIM-1 also increases the urinary KIM-1 in DN, verifying that KIM-1 can be a biomarker and a reliable predictor of diabetic kidney injury (122).

#### 3.2.4 Chromogranin A

The β-cell secretory granule protein, also known as chromogranin A, is a new autoantigen in T1D. A recent study identified chromogranin A as a forceful inducer of the reacting CD4^+^ T cells in the pathogenic process of T1D in NOD mice (123). However, studies on the function of chromogranin A in diabetic vascular complications and DKD are still insufficient.

## 4 T cells regulate inflammation in diabetic kidney disease through inflammatory cytokines

In DKD, the inflammatory cytokines secreted by T cells can cause the epithelial-to-mesenchymal transition and the extracellular matrix accumulation (124). In this section, we have elaborated on the mechanisms by introducing, summarizing, and comparing the inflammatory mediators in DKD, which may prove useful in future researches.

### 4.1 IL-1β

Based on the different encoding genes, IL-1, a classical chemokine, is divided into IL-1α and IL-1β. Both can bind to the primary receptor point of distinction (IL-1RI), while only IL-1β is secreted by T cells and macrophages (125). In diabetic metabolic syndrome, high glucose and oxidative stress can induce IL-1 activation, which occurs earlier than the pathophysiological manifestations. IL-1β production may be related to TNFR-Fas-caspase-8-dependent pathway in CD4^+^ T cell-driven autoimmune pathology (126). Moreover, IL-1β was also identified to cause endothelial cell damage in resistance arteries and affect the NADPH oxidase activation (127, 128). In addition, studies have shown that repressing IL-1β and its receptor can reduce systemic inflammation in patients with T2DM (128, 129).

### 4.2 IL-2

IL-2 can be produced by Th and kidney-derived MAIT cells. The function of the IL-2/IL-2R in renal dysfunction has been discussed in early studies, which has indicated that serum soluble IL-2R (sIL-2R) levels increase with a decrease in creatinine clearance (130). In the autoimmune diabetic NOD mice, two separate research groups have revealed that deficiency in IL-2 production or the responsiveness of Tregs to IL-2 may be associated with the development of the immune response (131, 132). Given its crucial role in the expansion and function of Tregs, IL-2 has been used to regulate tissue damage and limit the immune response following infection (133). Low-dose IL-2 selectively induces CD4^+^ CD25^+^ FOXP3^+^ Tregs in patients with CKD, and these Tregs limit the levels of proinflammatory Th1 and Th17 cells (133). In other mouse models of autoimmune diseases, such as C57BL/6 mice, CD4^+^ CD25^+^ Tregs are also induced by recombinant IL-2, thus preventing the progression of diabetes (134). Hence, it would be interesting to explore the effect of IL-2 on new therapeutic schedules for patients with DKD.

### 4.3 IL-4

IL-4, partly produced by Th2 and NKT cells, can expand the proliferation of activated T and B cells and regulate the differentiation of Th1 and Th2 cells (135). The role of IL-4 in DM remains controversial. Data investigation of Filipino patients suggested that the risk of T1D was partly determined by specific polymorphisms. The variability in promoters, coding sequences, and specific combinations of genotypes indicated that IL-AR of IL-4 and IL-13 were significantly associated with susceptibility to T1DM (136). In contrast, no significant change in IL-4 plasma levels between patients with T2DN and those without nephropathy was observed in a study (25). The IL-4 *rs2243250* polymorphism is irrelevant to DN in Slovenian patients with T2DM (137). As a result, the relationship between IL-4 and DN may depend on race, ancestry, geographical conditions, and national customs to some extent, which needs to be proven by more prospective evidence.

### 4.4 IL-6

IL-6 levels are significantly increased in the plasma of patients with DN patients than those with diabetes but without nephropathy (138). Furthermore, the *IL-6-174 G* allele was found to increase the occurrence rate of DN, confirming the correlation between IL-6 and DN (139). Similarly, a recent meta-analysis revealed the significance of different IL-6 polymorphisms in DN progression. The results showed that IL-6 *rs1800795, rs1800796*, and *rs1800797* were associated with DN, whereas IL-6 *rs2069837* and rs2069840 may be indifferent to the risk of renal complications in patients with T2DM (139).

Classically, IL-6 participates in the pathogenesis of DKD by various methods, including binding to the receptor IL-6R, sIL-6R trans-signaling pathway, and IL-6 autocrine signaling (140). IL-6 influences renal cells by relying on diverse signaling pathways. For instance, IL-6 facilitated mesangial expansion by infiltrating the mesangium, interstitium, and tubules, which has been observed in human renal biopsies (141). Second, the determination of samples from patients indicated that the width of the glioblastoma (GBM) was directly associated with fibrinogen and IL-6 levels in diabetic glomerulopathy (142). Moreover, the effects of IL-6 on diabetic renal injury may be due to increased insulin resistance and promotion of the inflammasome (143).

### 4.5 IL-9

IL-9 is mainly produced by a flock of T cells, such as Tregs and Th2 cells, and manipulates signaling pathways in renal immune diseases. For example, IL-9 protects against progressive glomerulosclerosis and tubulointerstitial fibrosis and regulates T cell-induced immune suppression in adriamycin-induced nephropathy and acute kidney injury (144, 145). Meanwhile, as characterized by T cell cytokines, IL-9 levels were evidently reduced in the diabetic group and positively correlated with the level of urea and microalbuminuria, which may be considered as an approach of T cells to address hyperglycemia damage (43).

### 4.6 IL-17

IL-17A can be produced by many types of CD4^+^αβ and γδ T cells, particularly Th17 cells (146). The IL-17 family is essential for the inflammatory response and includes six structurally related isoforms: IL-17A, IL-17B, IL-17C, IL-17D, IL-17E, and IL-17F. However, only IL-17A and IL-17F have unique functions in DN, whereas IL-17C and IL-17E are indifferent to DN (44).

Clinically, the decline in IL-17 levels is synchronous with the progression of DKD and is correlated with declining GFR (43, 147). IL-17A has been proven to not only trigger inflammatory signaling pathways associated with NF-κB downstream but also regulate the viability of T cells (147). However, another study reported the opposite result, indicating that IL-17A may increase the infiltration of inflammatory cells in renal tissue and blood pressure in mice (148)

As a potential immunologic therapeutic target for DKD, studies have suggested that intrarenal IL-17A1 CD41 T cells can be suppressed by mycophenolate mofetil, which is beneficial for treating albuminuria and tubulointerstitial fibrosis (41). All-trans retinoic acid was used to retain the capacity of Tregs to secrete IL-17 during hyperglycemia, implying an important role of IL-17 in DKD (149).

### 4.7 IL-22

In addition to studies on DKD, IL-22, mainly produced by Th22, was found to be downregulated in patients with DKD. Further observations indicated that the mechanism of IL-22 participating in inflammatory processes of DKD is intricate and comprehensive. Chen et al. demonstrated that IL-22 induced AMPK/AKT signaling and PFBFK3 activity, alleviating the level of dysfunctional mitochondria and the accumulation of reactive oxygen species (150). In addition, IL-22 can ameliorate renal fibrosis and attenuate microalbuminuria in DKDs (150, 151).

### 4.8 IL-35

Anti-inflammatory cytokine IL-35 is expelled by Tregs, regulatory B cells, and tolerogenic antigen presenting cells. Tregs were reported to infiltrate renal tissues to maintain homeostasis of the immune system in patients with diabetes and use IL-35 to intervene in the development of DKD (63).

### 4.9 INF-γ

Several studies have reported that T cells can be stimulated by high glucose concentrations and expedite IFN-γ production (83, 130, 152). Under conditions of high glucose concentrations, IL-12 can stimulate CD4 cells to produce IFN-γ. AGE-modified proteins bind to the receptor for AGE and T cells, inducing the synthesis and release of IFN-γ and accelerating inflammation of renal tissues (130).

### 4.10 TNF-α

As a synthetic product of T cells, TNF-α may be used as an indicator for evaluating DKDs (153). Many clinical studies have found that TNF-α is increased in the plasma and urine of patients with diabetes, leading to a higher risk of mortality, more serious macroalbuminuria, sodium retention, and renal hypertrophy (154–157). Specifically, TNF-α participates in the pathophysiological reaction in DN *via* diverse pathways, including altering intraglomerular blood flow, reducing glomerular filtration, inducing cytotoxicity to renal cells, and producing local reactive oxygen species (158–160).

## 5 Summary of other functions of T cells in diabetic kidney disease

**Figure 1:**
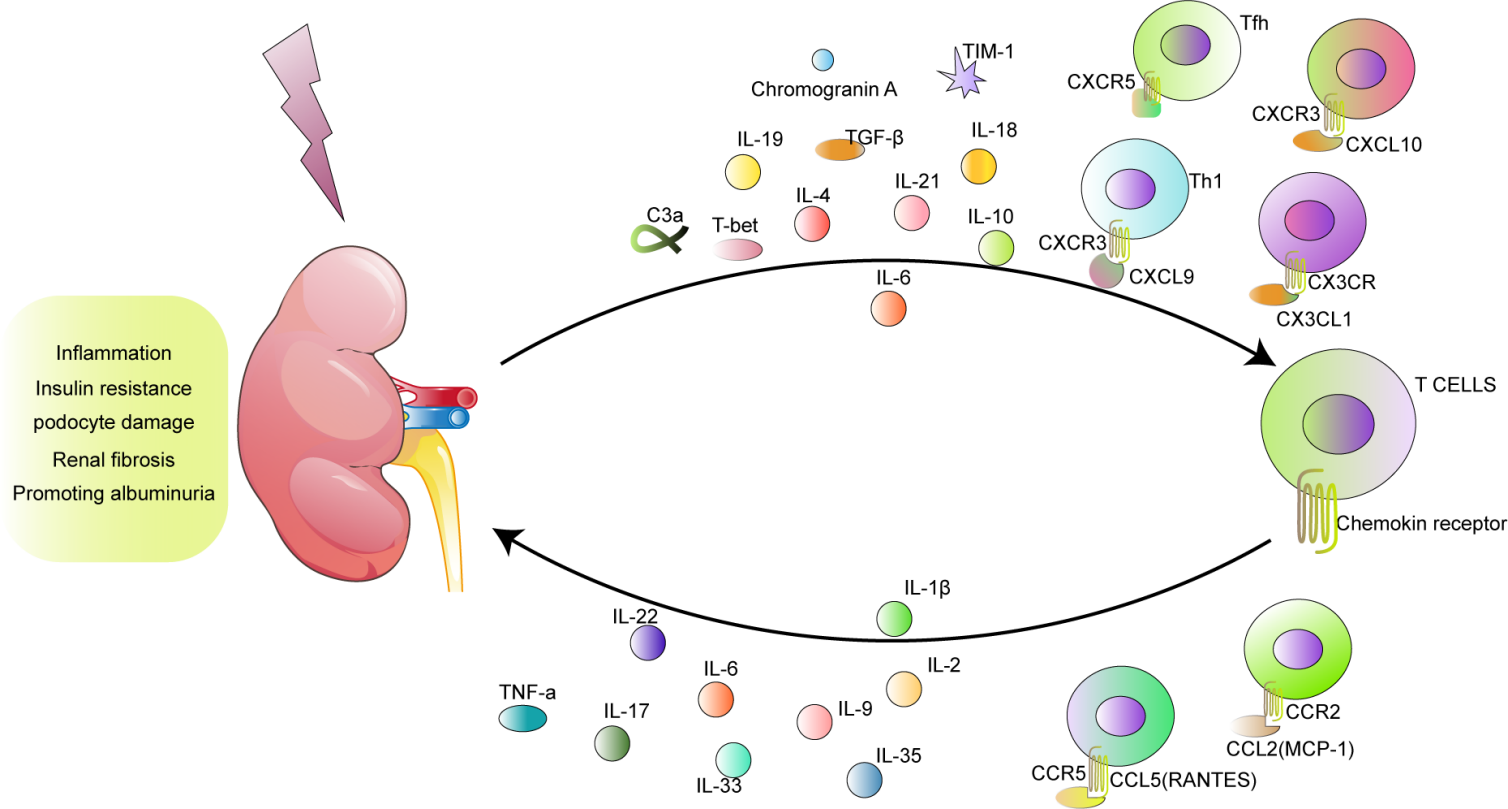
The model of T cells and their products participating in DKD.

Pathologically, hyperglycemia stimulates T cells to produce chemokines and cytokines that not only participate in the promotion of inflammation and activation of macrophages and endothelial cells but also damage renal function through different mechanisms. First, these proinflammatory molecules highlight the role of T cells in the process of insulin resistance. Second, T cells mediate the glomerular filtration barrier through podocytes. Third, T cells contribute to extracellular matrix deposition and the differentiation and proliferation of myofibroblasts. Ultimately, T cells lead to proteinuria and the development of DKD (**Figure 1**).

### 5.1 Function of T cells in insulin resistance

Insulin resistance is often regarded as a strong marker of DKD and is characterized by hyperinsulinemia and reduced insulin action, affecting many classical insulin-regulated pathways in the kidney and vasculature (161). For instance, the lack of insulin resistance in the kidney has been verified as an inducer of sodium retention, resulting in salt-sensitive hypertension. Podocyte insulin sensitivity is critical for glomerular alterations and disorders in DKDs (162, 163). In recent years, T-cells have been reported to improve glucose tolerance, enhance insulin sensitivity, and reduce weight gain in mouse models (164). However, a summary of T cells in renal insulin resistance is currently insufficient.

The relationship between insulin resistance and T cells has been described, involving Tregs, CD8^+^ T cells, Th cells, and MAIT cells. The depletion of Tregs leads to enhanced insulin resistance and impaired insulin sensitivity accompanied by albuminuria and glomerular hyperfiltration (165). In contrast, insulin sensitivity in DKD can be significantly rescued by adoptive transfer of CD4^+^ FoxP3^+^ Tregs in a murine model, resulting in less diabetic kidney damage (166). In addition, CD4^+^T cells in visceral adipose tissue have also been demonstrated to regulate insulin resistance and control glucose homeostasis in diet-induced obesity progression. When Th1 statically overwhelms CD4^+^ FoxP3^-^ Tregs, weight gain and insulin resistance are reversed (166).

Moreover, the depletion of CD8+ T cells has been reported to alleviate macrophage infiltration of CD8+ T cells. CD8 + T cells recruit macrophages to mediate insulin resistance and adipose tissue inflammation (65). Conversely, systemic insulin resistance is aggravated by adoptive transfer of CD8+ cells (167). In addition, Th1 and MAIT cells can regulate insulin resistance (77, 168).

As mentioned above, secretion and release of T cells produce proinflammatory cytokines that not only induce insulin resistance but also impair kidney function. IL-1, IL-6, IL-17, IL-33, GATA-3, and other proinflammatory cytokines also play important roles in renal insulin resistance. First, the blockade of IL-1 improves glycemia and β-cell secretory function; repression of the IL-6 receptor relieves diabetic renal injury and insulin resistance, and suppression of GATA-3 restores insulin sensitivity (129, 143, 169). In clinical investigations, TGF-β is positively correlated with insulin resistance markers, including fasting and postprandial glucose levels and HbA1c, whereas IL-17 is negatively associated with them (43). Additionally, studies have shown a serial decline in IL-33 levels in DN, resulting in an increased severity of insulin resistance and microalbuminuria (170). Overall, insulin resistance in DKD is closely associated with proinflammatory cytokines produced by T cells.

### 5.2 T cells and podocyte damage in DKD

Normal function and structural integrity of podocytes are essential for the occurrence of albuminuria and progression of diabetes (171). T cells and their production have been described as novel factors influencing podocytes in patients with diabetes (172). Therefore, podocytes may be regarded as an essential part of T cells that mediate pathological effects in DKD.

Firstly, CD28/B7 and cytotoxic T lymphocyte-associated antigen-4 (CTLA4) are critical for Th cells and podocytes. With regard to T-cell proliferation, differentiation, and survival, costimulatory molecules composed of CD28, B7-1 (CD80), and B7-2 (CD86) have been reported to play crucial roles (173). As a novel biomarker for podocyte damage, B7-1 is upregulated in podocytes under high glucose conditions. After activation, the CD28/B7-1 pathway mediates circulating T cells to aggravate podocyte damage (174, 175). Moreover, CTLA4 is a negative regulator of T cell activation, and genetic polymorphisms in CD28/B7/CTLA4 are related to susceptibility to T2DM (176). However, the fact that B7-1 was not inducible in podocytes in patients with DKD is contradictory; therefore, further investigation is required (177).

Researchers have also discovered that IL-17A is a characteristic proinflammatory cytokine in the serum and urine of patients with diabetes, and CD40 expression was observed to be increased in podocytes with DN (38, 147). The synergistic action of IL-17 and CD40L regulates the inflammatory response and mediates remodeling of glomerular sclerosis in DN.

Furthermore, podocyte damage is affected by TNF. Albuminuria is partly attributed to TNF-induced ABCA1 deficiency in podocytes. Studies have indicated that TNF is sufficient to cause free cholesterol-dependent podocyte injury through an NFATC1/ABCA1 dependent mechanism (155).

Podocyte apoptosis is triggered by CD8^+^ TRM cells. In db/db mice, the relative proportion of CD8^+^ TRM cells is remarkably increased under pathological conditions, and renal CD8^+^ TRM cells have cytotoxic effects on podocytes and enhance podocyte apoptosis (66).

### 5.3 T cells and renal fibrosis in DKD

Pathologically, fibrosis is one of the most fundamental characteristic mechanisms in the onset and progression of DKD, and renal T-cell infiltration is helpful for fibrosis. Therefore, hyperglycemia stimulates T cells and T cell-derived products, including IL-1, IL-6, IL-17, and IL-22, which are of central importance in progressive fibrosis in DKD (66).

Primarily, IL-1β induces proximal tubule damage and fibrosis in renal tubule interstitials (178). One study showed that IL-1β participates in the dysregulation of glycolysis and matrix activation, leading to tubulointerstitial fibrosis (147). In contrast, another report on CKD described the relationship between IL-1β and fibrosis initiation and progression (178).

Second, IL-6 trans-signaling may be a crucial factor in the development of renal fibrosis, thus influencing the width of the GBM in the pathogenesis of diabetic glomerulopathy (142, 179). Simultaneously, targeting IL-6 trans-signaling, Fc-gp130, could be a novel therapeutic strategy for renal fibrosis.

Moreover, IL-17 suppresses fibrosis *via* the STAT-3 and WAP domain protein pathways in models of T1D and T2D, and tubulointerstitial fibrosis can be rescued by suppressing intrarenal IL-17A1 CD41 T cells (41, 44). Furthermore, through the NLRP3/caspase-1/IL-1β pathway, IL-22 can reverse the overexpression of fibronectin, collagen IV, and extracellular matrix in mouse renal glomerular mesangial cells, thereby ameliorating renal fibrosis and proteinuria excretion in DN (150, 180).

Additionally, sphingosine 1-phosphate receptor 1 activation in T cells leads to fibrosis in normoglycemic conditions but exacerbates fibrosis in a model of STZ-induced diabetic cardiomyopathy (181).

### 5.4 T cells and albuminuria in DKD

#### 5.4.1 The quantity of T cells and albuminuria

To elaborate the internal relationship between T cells and albuminuria in DN, preliminary exploration was performed. Under STZ stimulation, only Rag1^(+/+)^ mice, which have mature T lymphocytes, had glomerular immunoglobulin deposition. However, Rag1^(-/-)^ mice, which lack kidney infiltration with T cells, were protected from albuminuria (172). Additionally, the degree of albuminuria is regulated by the number of T cells infiltrating the kidneys of DKD animals, and abatacept ameliorates DKD by blocking systemic T-cell activation (182).

However, there are also a series of contradictory reports. Absolute and percent T-lymphocytes were found to be relatively lower in patients with nephrotic proteinuria and long-standing insulin-dependent diabetes (183). In contrast, Another study showed that T cell-positive patients had a shorter duration of diabetes and lower albumin excretion rates than T cell-negative patients (84). Hence, the exploration and summary of the relationship between T cells and proteinuria in DN are significant.

#### 5.4.2 Types of T cells associated with proteinuria

In general, circulating CD8^+^ T cells and Tregs are considered the main types of T cells that are associated with albuminuria in DN. A cross-sectional study showed that the percentage of circulating CD8^+^ T cells was correlated with albuminuria in T2DM, indicating that systemic inhibition of T lymphocytes provides a new therapeutic direction for albuminuria in DKD (184). In addition, FoxP3^+^ Tregs exert a protective effect in the kidneys of diabetic mice, although it reduces glomerular hyperfiltration and albuminuria. Moreover, depletion of Tregs with anti-CD25 antibodies can accelerate the progression of albuminuria (165).

#### 5.4.3 Product of T cells and albuminuria

T cells regulate albuminuria through cytokines including IL-6, IL-9, TNF-α, IL-22, IL-33, and IL-233.

IL-6, associated with higher albuminuria, has been reported in db/db mice and patients with diabetes (143, 185). The IL-6 receptor antibody (tocilizumab) can reduce proteinuria and glomerular mesangial matrix accumulation. Furthermore, the levels of IL-9 and TNF-α are positively correlated with the levels of urea and microalbuminuria (43, 185, 186). Albuminuria may be caused by TNF-α via alterations in the glomerular capillary wall and an increase in albumin permeability.

In addition, studies on IL-22 support the hypothesis that cytokines drive proteinuria. IL-22 can alleviate mesangial matrix expansion and proteinuria in mice (151, 172). IL-33 also represses microalbuminuria in DKDs (170, 187). Intriguingly, the increase in IL-33 levels in DN is only associated with diabetes but not with kidney injury (188). Therefore, the exact role of IL-33 in DKD remains controversial.

Notably, a novel cytokine (named “IL-233”) possesses the activities of both IL-2 and IL-33 and protects against type-2 DN by promoting T-regulatory cells. Treatment with IL233 reduces hyperglycemia, plasma glycated proteins, and albuminuria, protecting mice from T2DN (189).

## 6 Promising novel therapies targeting T cells in DKD

Until now, the standard management strategy for DKD has prioritized strict glucose control and blood pressure with RAAS blockade. However, the therapeutic means are limited to stopping or reversing the progression of DN. Therefore, new drugs targeting the pathological mechanisms of DKD, such as T cells and their products, have drawn increasing attention (**Table 1**).

**Table 1:** The therapeutic methods targeting to T cells in DKD.

The therapeutic methods	Target T	Potential mechanism	Reference
Triptolide (TP)	Th cells	Regulating the Th1/Th2 cell balance in DN	152
miR-29b	Th1	Rescues renal inflammation and fibrosis	190
Enalapril	CD4^+^ and CD8^+^ T	Promoting expansion and activation of T cells	191
TNF-α and TCR therapy	T cells	Reverse the diabetic metabolic state in T1DM	193
Paroxetine	Tregs	Rescued the differentiation and the population of Tregs	194
CD3-specific antibody	Foxp3^+^	Promote the predominance of Foxp3^+^ cells over Th1 cells	166
Diabetea teame (DT)	Tregs	Promote the Treg/IL-17 ratio	195
Surfactin	Tregs CD8^+^ T	Increasing CD4^+^ CD25^+^ FOXP3^+^ Tregs Suppress CD8+ T cells	196
Recombinant human IL-2 and Tregs	Tregs	Regulating the trafficking and desensitization of Tregs in Type 1 Diabetes Increasing NK, MAIT, and CD8^+^ T cell	58, 133, 135
Adoptive Treg Immunotherapy	Tregs	Improved insulin sensitivity Down-regulating the ACR in DN	165, 197, 198
MSCs	CD8^+^T	Impairing the activation and proliferation of CD8^+^T Preventing the exacerbation of kidney injury	64, 199

### 6.1 Traditional drug therapy with T cells

First, there are some drugs that target the Th cells. Similar to triptolide, a well-known drug for DN, influences Th lymphocyte cells in rat models of DN by regulating the Th1/Th2 cell balance. DN is associated with the upregulation of Th1 cells and downregulation of Th2 cells; however, triptolide can alter this ratio in high-fat diets and STZ-induced rats (152). Concurrently, animal experiments have shown that miR-29b is a novel therapeutic agent for treating T2D that effectively rescues renal inflammation and fibrosis by inhibiting T-bet/Th1-mediated immune response (190).

Second, the expansion and activation of CD4^+^ and CD8^+^ T cells can be enhanced by Enalapril and γ-aminobutyric acid receptors in DKDs (191, 192).

In addition, combining anti-TNF-α therapy and the T-cell-specific antibody anti-TCR can reverse the diabetic metabolic state in a model of human T1D (193).

### 6.2 Tregs-targeted drugs

Researchers have discovered that administering drugs targeting Tregs can be beneficial in diabetic diseases. For instance, Paroxetine, a G protein-coupled receptor kinase 2 inhibitor, has been approved to rescue Treg differentiation and restore the population of circulating Tregs *in vitro* and *in vivo (* 194). In obese WT and ob/ob (leptin-deficient) mice, a CD3-specific antibody or its F(ab’)2 fragment can promote the predominance of Foxp3^+^ cells over Th1 cells (166).

Ethnopharmacological relevance Diabetea teame was verified to promote the Treg/IL-17 ratio in clinical settings, suggesting the protective effect of DT against diabetes-related complications in the long term (195). In addition, Surfactin, a bacillus-produced natural immunomodulator, could increase CD4^+^ CD25^+^ FOXP3^+^ Tregs while simultaneously suppressing T cell proliferation and downregulating the activated CD8^+^ T cells (196).

### 6.3 Recombinant human IL-2 and Tregs

IL-2 plays an essential role in the expansion of Tregs and can reduce tissue damage by limiting immune response. A single ultra-low dose of Aldesleukin (proleukin; recombinant human IL-2) has been demonstrated to regulate early altered trafficking and desensitization of Tregs in T1D (133). Simultaneously, low expression of mlL-2 also prevents the progression of diabetes by regulating Tregs in islets (135). Furthermore, combining low doses of IL-2 with exogenously administered Tregs leads to an increase in the number of Tregs, NK cells, mucosal associated invariant T cells, and clonal CD8^+^ T cells (58).

### 6.4 Adoptive Treg immunotherapy

Recently, expanded Tregs have been used to treat deficits in the number and suppressive activity of Tregs in immune-related diseases. Two separate research groups have explored adoptive Treg immunotherapy and demonstrated its safety, tolerance, and efficacy in patients with DM (197, 198). Bluestone et al. reported a phase 1 trial of adoptively transferred self-derived Tregs to repair or replace Tregs in patients with T1D. Simultaneously, adoptive transfer of CD4^+^ FoxP3^+^ Tregs significantly improved insulin sensitivity and decreased the albumin-to-creatinine ratio in DN (165).

### 6.5 Mesenchymal stem cells

In the last decade, MSCs have been widely used to treat DN. Intriguingly, MSC-CM pretreatment reduced CD8^+^ T cell priming and proliferation capacities in the kidneys of DN rats (64). Furthermore, MSC transplantation not only impairs the activation and proliferation of CD8^+^ T cells but also prevents the exacerbation of kidney injury, providing a new insight into the treatment of DN (199).

## 7 Conclusion

With the increase in the number of patients suffering from DKD, exploration of the function of T cells in DKD is increasingly important. After circulating T cells are recruited into the renal tissue or T cells are amplificated, differentiated, and activated in the kidney, T cells play protective or pathogenic roles through multiple pathways, including influencing insulin resistance, mediating podocyte damage, participating in fibrosis, and regulating proteinuria (**Table 2** and **Figure 1**).

**Table 2:** The fundamental function of T cells in DM and DN.

Regulatory factors	T cells population	Cytokine secretions	Key finds in DM and DN	Reference
STAT4↑ STAT1↑ T-bet↑ TIM3↓ IL-10↓ IL-4↓	Th1	IL-2↑ IFN-γ↑ TNF-α↑ T-bet↑	Activating macrophages Associated with proteinuria and creatinine clearance	17–25
GATA-3↑	Th2	IL-4↑ IL-5↑ IL-9↑ IL-10↑ IL-13↑	Suppress Th1 cell activation	26–33
JAK/STAT↑ STAT3↑ IL-2↓ STAT5↓ TGF-β↑ IL-1β↑ IL-6↑ IL-23↑	Th17	IL-17↑	Aggravating diabetic renal Regulating Th17/Th1 and Th17/ Treg Increase inflammatory Correlated with GFR	26, 34–46
	Th3	TGF-β↑		47
	Th9	IL-9 ↑	Associated with podocyte injury and ACR in T1DM	48
	Th22	IL-22↑		49
IL-6 and IL-21↑	Tfh	IL21↑	Manipulate the level of estimated creatinine, urea and urinary protein level, Fasting and postprandial blood glucose, Hemoglobin A1c in diabetic nephropathy	50–55
IL-2↑	Tregs	IL-2↑ IL-17↑ IL-10↓ IL-35↓ TGF-β↓	Maintaining the balance in the anti-inflammation and anti-inflammation in diabetes condition Limit the pro-inflammatory Th1 and Th17 Lessening glomerular hyperfiltration and albuminuria	56–63
	CD8+ T		Recruiting macrophages Ameliorating systemic insulin resistance Promoting podocyte injury Accelerating glomerulosclerosis	64–66
	NKT		Kidney damage through FasL pathway Taking part in the exacerbation of DM	67–72
	γδ T		Upregulated, but the mechanism is unknown	73
	MAIT	IL-2 GM-CSF IL-17	Influence the insulin resistance Promoting the level of HbA1c	74–78

As **Table 1** showed, based on the significant functions of T cells and cytokines, the application of T cell-associated therapies in DKD has been attempted and preliminary achievements have been made. Promising studies on T cell biology will unquestionably contribute to a more profound understanding of DKDs, highlighting the need to identify new therapeutic approaches.

## Author contributions

YLi and YLv conceived and drafted the review article. TZ, TH and YLa created the model figure. QS and YLi prepared the tables. ZK, YG, SL were involved in the compilation of the references. MY and YLu revised the manuscript. All authors contributed to the article and approved the submitted version.
